# Contemporary issues concerning informed consent in Japan based on a review of court decisions and characteristics of Japanese culture

**DOI:** 10.1186/1472-6939-15-8

**Published:** 2014-02-04

**Authors:** Sakiko Masaki, Hiroko Ishimoto, Atsushi Asai

**Affiliations:** 1 Department of Bioethics, Kumamoto University Graduate School of Life Science, Kumamoto, Japan

**Keywords:** Informed consent, Japanese culture, Ethical principles, Ethical issues, Misuse, Healthcare professionals

## Abstract

**Background:**

Since Japan adopted the concept of informed consent from the West, its inappropriate acquisition from patients in the Japanese clinical setting has continued, due in part to cultural aspects. Here, we discuss the current status of and contemporary issues surrounding informed consent in Japan, and how these are influenced by Japanese culture.

**Discussion:**

Current legal norms towards informed consent and information disclosure are obscure in Japan. For instance, physicians in Japan do not have a legal duty to inform patients of a cancer diagnosis. To gain a better understanding of these issues, we present five court decisions related to informed consent and information disclosure. We then discuss Japanese culture through reviews of published opinions and commentaries regarding how culture affects decision making and obtaining informed consent. We focus on two contemporary problems involving informed consent and relevant issues in clinical settings: the misuse of informed consent and persistence in obtaining consent. For the former issue, the phrase "informed consent" is often used to express an opportunity to disclose medical conditions and recommended treatment choices. The casual use of the expression "informed consent" likely reflects deep-rooted cultural influences. For the latter issue, physicians may try to obtain a signature by doing whatever it takes, lacking a deep understanding of important ethical principles, such as protecting human dignity, serving the patient’s best interest, and doing no harm in decision-making for patients.

There is clearly a misunderstanding of the concept of informed consent and a lack of complete understanding of ethical principles among Japanese healthcare professionals. Although similar in some respects to informed consent as it originated in the United States, our review makes it clear that informed consent in Japan has clear distinguishing features.

**Summary:**

Japanese healthcare professionals should aim to understand the basic nature of informed consent, irrespective of their attitudes about individualism, liberalism, and patient self-determination. If they believe that the concept of informed consent is important and essential in Japanese clinical settings, efforts should be made to obtain informed consent in an appropriate manner.

## Background

Beauchamp and Childress argued that virtually all codes of medical ethics and institutional regulations should require physicians to obtain informed consent from patients prior to substantial interventions, with the protection of patient autonomy as the primary justification for this requirement. They also claimed that informed consent is an individual’s autonomous authorization and postulated seven structural elements [1], including threshold elements (competence to understand and decide; voluntariness in deciding), information elements (disclosure of material information; recommendation of a plan; understanding of the information and recommended plan), and consent elements (decision in favor of the plan; authorization of the chosen plan) [1]. We think that these elements are quite clear and comprehensive, and could provide a useful framework for the critical review of various contemporary issues surrounding informed consent acquisition in Japan.

The concept of informed consent received a great deal of attention during the 1980s in Japan. In 1990, informed consent was translated into Japanese as "setsumei to doi" (back-translated as "explanation and consent"). This Japanese translation, however, carries a connotation that informed consent is a duty owed to patients and does not properly purport the notion that informed consent is a patient’s right [2]. In other words, the Japanese translation fails to grasp the "consent elements" of the framework described above. Currently in Japan, informed consent is often obtained without the patient’s understanding, physician’s recommendation, or adequate time to think [3]. In Japan as well as in other countries, many difficult issues regarding patient self-determination and acquisition of informed consent remain even after an ethical norm to obtain informed consent from patients in clinical settings and for research projects has been developed and established. They include compulsive interventions, treatment decisions for incompetent patients or minors, and issues surrounding treatment refusal [4,5].

In this paper, we discuss current situations and cultural characteristics concerning informed consent in Japan to outline the problems that we think are common and relevant. First, we review five court decisions related to informed consent and information disclosure. Next, we discuss the characteristics of Japanese culture by reviewing published opinions and commentaries. Then, we describe two contemporary issues concerning informed consent in current clinical settings in Japan: misuse of informed consent and persistence in obtaining consent. Finally, we present our opinion on current situations surrounding informed consent in Japan. Our focus is on informed consent in clinical settings; we do not address informed consent in research settings.

## Discussion

### Court decisions concerning informed consent in contemporary Japan

In the past three decades, the Japanese Supreme Court has set forth decisions in four cases concerning truth-telling and informed consent, and one district court considered a case about the necessity of disclosure to families of patients. The first case concerned disclosure of a cancer diagnosis. A physician failed to inform a patient that she had gall bladder cancer, but instead told her that she had a gall stone that required inpatient care. However, the patient did not come back to the hospital and, as a result, the physician did not inform either the patient or the patient’s family. In 1995, the Japanese Supreme Court concluded that a physician does not need to disclose a cancer diagnosis on the ground that the physician can overlook a patient’s right to self-determination, if, in their judgment, the actual diagnosis could have an adverse impact on the patient [2]. In this case, the principle of non-maleficence was prioritized over respect for patient autonomy.

In the second case, the Japanese Supreme Court considered whether a physician is obligated to inform his patient about a conservative, yet non-established, breast cancer treatment [4]. At the time, mastectomy and conservative treatment were the only two options available for the patient. The physician advised the patient that while the conservative treatment for breast cancer had been implemented, this method was not yet fully and accurately understood. The physician also told the patient that her breast would be totally removed, but the pectoral muscle would remain. Before surgery, the patient gave the physician a letter outlining the complex sentiments of a woman who had been diagnosed with breast cancer and faced the choice of continuing to live and having her breast removed.

In 2000, the Court stated that there are instances in which a physician is under an obligation to provide an explanation, even for non-established treatments. In this case, the conservative treatment had been implemented at multiple medical institutions, and the results had been positively assessed by participating physicians. The treatment may have been suitable for the patient, particularly if the patient showed a strong interest in the applicability of the treatment to her situation [4]. The Court added that mastectomy for breast cancer is an operation that involves removal of the breast and can seriously affect the patient’s mental and psychological state, as it changes her appearance and impacts quality of life. Thus, physicians are obligated to explain the conservative treatment for breast cancer as an alternative treatment before the patient decides on surgical removal of breast tissue. This requirement is even more pronounced in this case compared to other operations that do not have such an impact on the patient’s appearance and/or quality of life. Some argue that the physician should have given the patient an opportunity to determine the course of treatment and not deprive her of information about an alternative treatment, only because the treatment had not been established [4].

In the third case, the Japanese Supreme Court considered whether physicians should have given a blood transfusion during surgery to a patient who had joined the Jehovah’s Witnesses and firmly refused blood transfusions [5]. Based on the hospital policy regarding surgery on patients who belong to the Jehovah’s Witnesses, the hospital would respect the patient’s intention to refuse blood transfusions and refrain from providing transfusions to the extent possible. However, in the event that there was no means of saving a patient’s life other than through a blood transfusion, the hospital would give the patient a transfusion irrespective of whether the patient or his/her family had approved such treatment. During the operation to remove the patient’s tumor, the amount of bleeding reached more than two liters. Determining that it was highly unlikely that the patient’s life can be saved without a blood transfusion, the patient was given a transfusion during surgery. In 2001, the Court determined that when a patient expresses an intention to refuse any medical treatment involving a blood transfusion because it is against their religious beliefs, the right to make such a decision must be respected as the patient’s personal right. It would have been reasonable for the physician to explain to the patient that irrespective of the patient’s or family’s approval, it is the hospital’s policy to give a blood transfusion if such a transfusion is required to save the patient’s life. The patient could then have decided whether or not to be operated on at that hospital [5,6]. As the physicians failed to provide a sufficient explanation, they should be held responsible for violating the patient’s personal rights, because she was deprived of her right to decide whether or not to be operated on. In this sense, they are liable to compensate her for the mental distress she suffered.

In the fourth case, a physician continued seeing a patient with terminal lung cancer in an ambulatory care clinic without disclosing the cancer diagnosis to either the patient or the patient’s family. The family was later told at a different hospital that the patient suffered from terminal lung cancer, and the patient subsequently died. In 2002, the Supreme Court concluded that if a physician does not disclose a cancer diagnosis to a patient, the physician is under an obligation to contact the patient's family directly and disclose the diagnosis, and discuss whether or not the diagnosis should be told to the patient [2,7].

In the final case, a physician informed a patient that he had prostate cancer and recommended that he receive specialized medical care at another medical institution [7]. However, the patient did not want to undergo further aggressive treatments and continued to see the same physician. The patient died of cancer three years later. Bereaved family members did not know about the patient’s diagnosis and sued the physician, complaining that he had a duty to inform the family regarding the diagnosis. In 2007, the Nagoya District Court concluded that a physician has no obligation to inform a patient’s family of a cancer diagnosis as long as the patient is clearly informed, that it should be the patient who decides whether or not to undergo a treatment, and that if the physician fulfilled his/her responsibility to provide sufficient explanation to the patient and the latter made the treatment decision him/herself, the physician can be considered to have performed his/her legal duty adequately [7].

Thus, currently, physicians in Japan do not have a legal duty to inform patients of a cancer diagnosis. They do not have a legal duty to conceal a cancer diagnosis from the patients, either. However, physicians have a legal duty to inform the family if the patient is not notified. Conversely, the physician has no legal duty to tell the patient’s family if the physician has already informed the patient [7]. Historically in Japan, there was no requirement for physicians to disclose the true diagnosis to a person suffering from cancer; they only notified family members. Even today, some physicians are reluctant to practice full disclosure to patients when it comes to a cancer diagnosis. This may represent one of the Japanese medical cultures reminiscent of past practice.

Treatment refusal based on religious faith is supported based on the personal rights of patients. Physicians have a legal duty to inform a patient of as many alternatives as possible, and court decisions demanding compensation for damages based on inadequate information disclosure continue. However, a right to self-determination at the end-of-life has not been legally or socially established. When death is not imminent, the right to refuse life-sustaining treatment has not been considered at all in Japan. The certainty of impending death appears to have become the main prerequisite for respecting patient decisions. In addition, no court, legislation, or guidelines have ever presented a clear time limit or precise definition of "impending" [8].

### Literature review regarding the characteristics of Japanese culture

In order to examine issues surrounding informed consent in Japanese clinical settings, it is necessary to understand the characteristics of Japanese culture as well as recent court decisions and incidents. Cultural peculiarities in Japan may explain some of the actions relevant to informed consent in Japanese clinical settings. In the following section, we review published papers on Japanese cultural characteristics in the context of healthcare without using stereotypical or careless assumptions [9]. Dividing West and East equally would naturally be too simplistic. There are various countries in both the East and West, with differences among individuals, generations, families, regions, and current events even within the same culture. Some aspects change over time while others remain constant. Certain aspects may be common across diverse cultures. Individuals can also significantly change their cultural perspectives during their lives. Taking this into consideration, we briefly review and discuss Japanese culture below.

#### Harmony without overriding principles

Izawa argued that the idea that harmony is the greatest virtue enshrined in the Constitution established by Prince Shotoku in Japan (604 AD) is still deeply rooted in modern Japan [10]. The Constitution demands that people must not conclude important matters alone and that serious matters must be discussed by a group. Matsuda suggests in his 2010 paper that Japanese culture has particular characteristics, including vagueness that does not draw a definitive line between right and wrong, behavior directed by others according to heteronomy rather than autonomy, avoidance of conflicts and confrontation, and indirect expression to avoid hurting others. He also points out that Japanese tend to be non-analytic [11]. Asai claimed that a fundamental principle in Japanese society is to not give priority to one principle over another, making it difficult to reach a specific conclusion on ethical dilemmas or conflicting situations [9].

#### Tacit understanding requiring "telepathy"

Asai suggested that "telepathy" (tacit understanding) is the preferred communication style in Japanese healthcare settings, which has been perpetuated over many years [12]. Those who regard tacit understanding as being a natural form of human communication might demand that others understand what they have in mind without being told. In addition, Japanese people are thought to be traditionally burdened with the trilemma of "won’t speak," "won’t decide," and "won’t think" when it comes to ethical issues [13].

#### Cultural relativism

It has been suggested that Japanese people do not pay attention to universality and consistency in decision-making and that they tend to be partial to relatives and acquaintances as a consequence. Furthermore, cultural relativism seems to be prevalent in justifying certain decisions on the grounds that "We are Japanese" or "We live in Japan now" [12]. A paper regarding self-determination in death argued that some Japanese commentators thought that self-determination is not desired, does not apply to serious matters such as life and death, and that respecting self-determination in death inflicts harm on others [14]. Tanida also pointed out that, in Japanese thought, a person does not exist as an individual, but as a member of the family, community, or society. In this society, an act is ‘good’ and ‘right’ when it is commonly done; it is ‘bad’ and ‘wrong’ if nobody else does it [15].

#### Non-individual-oriented education and psychology

Sasaki argued that Japanese are trained to be cooperative rather than autonomous and independent since childhood and that acting different from family members or neighbors may require a significant amount of energy and result in psychological distress, such as uneasiness and guilt. The mentality of "Follow your child when you grow old" in Japanese society has been suggested, and the elderly sometimes entrust decisions about their healthcare to family members. Families of elderly patients together with healthcare professionals might naturally take the initiative regarding healthcare decisions for the patient without their explicit consent. Thus, a family-centered or group-centered approach has been predominant in Japanese clinical settings [16]. Patience and modesty are considered virtues, and disobedience to group decisions is considered unacceptable.

Tamura also discussed dominant problematic mentalities. In Japanese society, one often feels the need to consider family members’ thoughts and feelings when making a decision. There is often internal or external pressure to prioritize family members’ opinions over those of the individual. Even when the family says that it is the person’s decision, he or she may still feel that the feelings of other family members should be prioritized [17]. Ignoring one’s true desire is sometimes considered a virtue in Japan. From childhood, people are taught to respect others, especially parents, teachers, authorities, and older people [17]. A follow-the-crowd mentality and unassertiveness are often observed among Japanese people, as well as a tendency to eschew free thinking and discussion regarding individuals, hierarchy, and conventionalism at the workplace [13].

#### Interdependence

The literature view suggests the presence of traditional norms, such as interdependence, entrusting others, and filial duties [18,19]. The well-known Japanese psychiatrist Doi argued that Japanese tend to expect others to consider what they need and unconsciously require others to act in their best interest [20]. A commentator claimed that the predominantly Japanese idea to entrust important decisions to others could have its ideological origin in Buddhism [21]. Therefore, the Japanese mental tendency pointed out by Doi could result in patients depending on their physicians and other healthcare professionals when decisions must be made regarding medical care. However, when things go wrong, patients and their families may criticize healthcare professionals and accuse them of being solely responsible for the poor outcome.

#### Different implications with the same appearance

Despite the strong influence of Western cultures, imported rules and concepts from the West may change in their implications, functions, and even goals without changing their appearances and thus become Japanesque in Japanese society [11,12]. Even though the names of concepts and principles may remain the same, they are likely to be similar yet different, and this likely reflects the strong, mixed influence of Shintoism, Buddhism, and Confucianism.

### Misuse of informed consent and persistence in obtaining consent

In the following section, we discuss two contemporary problems concerning informed consent in Japan and consider why these situations occur. As clinicians involved in patient care in several Japanese medical institutions, we unanimously and firmly believe that these problems currently exist, although we cannot present sufficient empirical data based on well-designed descriptive studies and do not claim that these issues are a universal phenomenon across Japan. We think that some of the problems can be ascribed to Japanese cultural characteristics, as discussed below.

#### Misuse of informed consent

There are several expressions concerning informed consent frequently used by Japanese healthcare professionals, especially physicians, such as "I will/do (provide) informed consent," "I go to informed consent," "I indicated informed consent was provided," and "There will be informed consent." For example, some physicians might say "I have just done (provided) informed consent to my patient." In the first three cases, the subject is the physician. The subject in the fourth case is unknown [9]. Although no report has yet to describe the variable use of these expressions, some academic societies and hospitals have official homepages set up regarding this issue, and we found a textbook that uses the expression, "We (I, you, or no subject) do informed consent" [22-24].

According to the basic idea of informed consent, these expressions do not make sense, and the expression should be "A physician obtains informed consent from his or her patient," in which the subject of the sentence is the physician. The process of obtaining informed consent from the patient includes the physician disclosing relevant information to the patient, presenting recommendations to the patient, answering the patient’s questions, and discussing alternatives together. The function of physicians should never be directly related to providing consent. It goes without saying that it is the patient who gives informed consent. A patient gives his or her physician informed consent and the physician obtains it from the patient. It is illogical that the physician ‘does (provides)’ or ‘places’ consent as part of the process.

However, as far as we know, it is not uncommon in Japan for physicians to use expressions such as "I will do (provide) informed consent." The phrase "informed consent" is often used to express an opportunity to disclose medical conditions and recommend treatment choices. For example, a physician could express dissatisfaction to a colleague by saying "I have already carefully done (provided) informed consent" after a patient asks several questions about treatment plans. In the United States, some physicians might have regarded the process of consent acquisition as merely explaining to patients the nature of their medical conditions together with a recommended treatment plan in the 1980s, when disclosure was considered the primary (and perhaps sole) element of informed consent [25].

Why does the misuse of informed consent occur and continue in Japan? We suspect that the casual use of words regarding informed consent reflects deep-rooted cultural influences in Japanese society [26]. The idea that the patient makes the final decision about his or her medical care has not been recognized in Japanese clinical settings [2]. Only the name and formalities related to informed consent remain the same. The underlying spirit of informed consent may be lost and replaced by the traditional attitude that physicians decide what the patient should do. Japanese informed consent, with the physician as the subject, has possibly become similar, yet different, than intended.

We suspect that the reason physicians make the decisions and apply medical care on behalf of the patient in clinical settings is because they do not know the history behind the idea of informed consent, as the idea was imported a few decades ago. It is also argued that Japanese physicians might consider such knowledge unnecessary because of Japanese exceptionalism, which is deeply pervasive whether they realize it or not. According to LaFleu, Japanese exceptionalism implies that Western philosophies and practices do and should have only limited applications in Japan, and that Japan has a different set of social, philosophical, and religious traditions [27]. This justifies the limited adoption of Western bioethical principles and practice, with informed consent among them. In addition, many Japanese patients might not always desire self-determination due to their Japanese, heteronomous character [11].

Some Japanese physicians may assume that they know what their patients want through tacit communication and take for granted that their patients may prefer treatment options that prioritize medical benefits. Japanese patients may also expect that their physicians, who are medical authorities, will consider their needs and consciously or unconsciously act in their best interest. We suspect that the nature of informed consent in which the subject is the physician has changed and lost the fundamental goal of respecting patient autonomy, even if the formalities remain the same. That is, it is similar to, yet different from, authentic informed consent. We also suspect that physicians who incorrectly use the phrase "informed consent" do not anticipate that their patients might refuse treatment because, in their minds, they lead the informed consent process.

Another possible reason is that the term "informed consent" has been directly incorporated into Japanese phases (i.e., it is not translated into Japanese but rather used as a Katakana word [Japanese-English]). Therefore, some physicians might misunderstand the meaning of it and say, "I will do the informed consent (in-form-con-sent wo suru)," or "I will provide informed consent to a patient," rather than "I will obtain informed consent."

#### Obtaining informed consent no matter what

In an aging Japanese society with a growing number of nuclear families and elderly who live alone, acquiring consent for incompetent elderly patients with no relatives has become a major perplexing issue in the clinical setting. An increasing number of healthcare professionals and hospital managers have confronted situations in which they cannot find anyone to provide informed consent for medically necessary interventions for elderly patients who lack the mental capacity to make decisions [28]. For example, the Japanese psychiatrist Sato commented in his case report that obtaining informed consent for life-saving treatments in patients with dementia who live alone is often problematic [29].

One of the authors has actually been involved in several discussions on this matter as a member of ethics committees at different hospitals. There appears to be a psychological tendency among healthcare teams and hospital managers to assume that someone must provide consent for medical treatment of incompetent patients with no relatives in every situation without exception. They may desperately try to obtain consent for a demented patient with no relatives from anyone available, including staff from the nursing home where the patient resides, an administrative officer in their community, or a completely estranged relative who has not talked to the patient for several decades. However, these people cannot serve as appropriate surrogate decision-makers. Healthcare teams often try to get someone’s signature on the consent form, no matter whose consent it is. It would be ethically and legally acceptable for healthcare professionals to conduct medical interventions without consent, if the intervention was beneficial to the patient and considered socially acceptable [30].

Why do these situations arise in Japanese clinical settings? The exact answer is unclear, and it seems impossible to explain solely based on Japanese cultural disposition. Yet, one possible reason is that healthcare professionals and those involved want to use a signed consent form in the event a problem occurs. Those who regard a signed consent form as a pardon may think that their responsibility for subsequent issues can be reduced by the fact that someone consented to undergo an intervention or that the person who provided consent should be partially responsible for whatever happens to the patient [3,31].

We argue that healthcare professionals, especially physicians, who try to obtain someone’s signature by doing whatever it takes lack a deep understanding of important ethical principles, such as protecting human dignity, serving the patient’s best interest, and doing no harm in decision making for patients. The idea that patients should be treated with their best interest in mind, even if physicians cannot respect the patient’s autonomy and obtain their consent, appears to be completely lost among healthcare professionals in Japan. It can be argued that the phenomenon of obtaining informed consent by doing whatever it takes occurs because those concerned do not logically consider medical ethics and simply do what they are told by superiors (i.e., they should always get a signature on the consent form) without real conviction about the purpose of informed consent. This is the product of social formalism without logical comprehension: an act is ‘good’ and ‘right’ when it is commonly done; it is ‘bad’ and ‘wrong’ if nobody else does it [15].

## Summary

In this paper, we discussed a number of decisions set forth by the Japanese Supreme Court concerning informed consent, characteristics of Japanese culture, and two contemporary issues that are common and relevant in Japan, including misuse of informed consent and persistence in obtaining consent. We suggested that there is a misunderstanding regarding informed consent and lack of a comprehensive understanding of ethical principles among healthcare professionals in Japan. It can be argued that informed consent in Japan is similar to, yet different from, original informed consent that was born in the US.

Healthcare professionals may fail to grasp and appreciate differences in individual values, diversity of personal world views, and ethical principles underlying the concept of informed consent. These failures could be related to the aforementioned characteristics of traditional Japanese culture. We argue that Japanese healthcare professionals should make efforts to understand the basic nature of informed consent, irrespective of their attitudes about individualism, liberalism, and patient self-determination, and obtain informed consent in an appropriate manner if they believe that the concept of informed consent is important and essential in Japanese clinical settings.

Finally, as both educators and researchers in bioethics, we believe that bioethics education that takes into consideration Japanese cultural and social contexts may be necessary for healthcare professionals in Japan in order to prevent the misuse of informed consent and misunderstanding of important ethical principles. Bioethics education which considers Western bioethics and Japanese culture in a complementary manner may also help in efforts toward the appropriate use of informed consent in clinical settings [32]. For these efforts to be effective, Japan’s long history and strong cultural tradition should not be ignored.

## Abbreviations

UNESCO: United Nations Educational, Scientific, and Cultural Organization.

## Competing interests

Sakiko Masaki, Hiroko Ishimoto, and Atsushi Asai declare that that they have no competing interests.

## Authors’ contributions

SM, HI, and AA have equally contributed to the conception, design, acquisition, and interpretations of references; drafting the manuscript and revising it critically for intellectual content; and have given final approval of the version to be published.

## Pre-publication history

The pre-publication history for this paper can be accessed here:

http://www.biomedcentral.com/1472-6939/15/8/prepub
